# Pediatric Behçet's Disease

**DOI:** 10.3389/fmed.2021.627192

**Published:** 2021-02-03

**Authors:** Mehmet Yildiz, Fatih Haslak, Amra Adrovic, Sezgin Sahin, Oya Koker, Kenan Barut, Ozgur Kasapcopur

**Affiliations:** Department of Pediatric Rheumatology, Cerrahpasa Medical School, Istanbul University-Cerrahpasa, Istanbul, Turkey

**Keywords:** Behçet's disease, children, clinical features, epidemiology, classification, treatment, pediatric, juvenile

## Abstract

Behçet's Disease (BD) is a systemic vasculitis firstly described as a disorder causing aphthous lesion in oral and genital mucosae and uveitis. The disease has an extremely unique distribution characterized by the highest incidence in communities living along the historical Silk road. Although our understanding of the etiopathogenesis of BD has expanded over time, there are still lots of unidentified points in the underlying mechanisms of the disease. The accepted opinion in the light of the current knowledge is that various identified and/or unidentified infectious and/or environmental triggers can take a role as a trigger in individuals with genetic susceptibility. Although the disease usually develops in young adulthood, it is reported that about 15–20% of all Behçet's patients develop in childhood. Pediatric BD differs from adult BD not only with the age of onset but also in the frequency and distribution of clinical findings, disease severity and outcome. While gastrointestinal system involvement, neurological findings, arthralgia and positive family history are more common in children, genital lesions and vascular lesions are more common in adult patients. In addition, a better disease outcome with lower severity score and activity index has been reported in children. The diagnosis of the disease is made according to clinical findings. It can be challenging to diagnose the disease due to the absence of a specific diagnostic test, and the long time interval from the first finding of the disease to the full-blown disease phenotype in pediatric cases. Therefore, many classification criteria have been proposed so far. The widely accepted ones are proposed by the International Study Group. The new sets of classification criteria which is the only one for pediatric BD were also developed for pediatric cases by the PEDBD group. The primary goal for the treatment is preventing the organ damages by suppressing the ongoing inflammation and forestalling the disease flares. The treatment of the BD can be onerous due to its multisystemic nature and a multidisciplinary approach is essential for the management of the patients. In this review article, the definition, clinical findings, epidemiology, etiopathogenesis, and treatment will be discussed.

## Introduction

Behçet's Disease (BD) is a systemic vasculitis with unique geographic distribution around the historical silk road. It is firstly defined as a disease causing recurrent oral and genital aphthae with uveitis by Hulusi Behçet (1). With the expanding knowledge about the genetic basis, etiopathogenesis and clinical findings, now we all know that BD has more extensive findings than the definition of Hulusi Behçet. It is a systemic vasculitis that can affect any size and type of the blood vessels and involve nearly all of the organ systems including gastrointestinal, nervous, musculoskeletal and cardiovascular systems (2, 3). Although our understanding of the underlying mechanism of BD is expanding day by day, the etiopathogenesis and the immunogenic background of the disease could not be fully explained and remain unclear. The widely accepted opinion in the light of the current knowledge is that various identified and/or unidentified infectious and/or environmental triggers may take a role as a trigger in individuals with genetic susceptibility. Although the disease usually develops in young adulthood (between the second and fourth decades of life), it is reported that about 15–20% of all Behçet's patients develop in childhood (3, 4). Pediatric BD differs from adult BD not only with the age of onset but also in the frequency and distribution of clinical findings, disease severity and outcome. In addition, pediatric onset disease usually starts with incomplete clinical phenotype and the development of a full-blown disease phenotype takes longer in pediatric patients (5–8). The diagnosis of BD is established according to the clinical findings due to the lack of a specific diagnostics test for the diagnosis of BD. Thus, several diagnostic and/or classification criteria have been proposed for adult-onset BD so far and the widely used are International Study Group classification criteria (9–11). The only classification criteria prosed for pediatric-onset BD is the classification criteria by The Pediatric Behçet's Disease group (12). Due to the extensive distribution of the disease among the various organ systems, the management of BD should be made with a multidisciplinary approach. In this review article, the definition, clinical findings, epidemiology, etiopathogenesis, and treatment of the pediatric Behçet's Disease will be discussed.

## Epidemiology

The prevalence of BD varies between geographic regions and the highest prevalence has been reported in communities living along the historical Silk road. Thus, the disease is called “the Silk road disease” by some of the physicians (13). The pooled prevalence of BD is reported as 10.3 per 100.000 population (14). While the highest prevalence worldwide is reported from Northern Jordan (664/100.000 population) which is followed by Turkey (600/100.000 population), the lowest prevalence was reported in Scotland (0.3/100.000 population) (15–18). The frequency of BD is affected by not only geographic region of residence but also ethnicity. The frequency of BD among immigrant population in Europe is reported as higher than in native population, but lower than in people who lives in their hometown (19, 20). There is no exact data about the prevalence of pediatric BD. It is reported that 4–26% of patient with BD have pediatric onset (2, 21).

Mean age at the onset of pediatric BD ranges from 4.9 to 12.3 years and delay in diagnosis is about 3 years in reported pediatric cohorts (3, 22). Similar to adult onset BD, pediatric onset Behçet's Disease is also seen equally in both genders (4). The frequency and severity of the clinical manifestations vary between genders. In general, it seems that males have more severe disease course than females (23–25). While severe uveitis and vascular disease are more common in males, genital aphthae, and erythema nodosum are more common in girls (2, 25, 26). It is also shown that the frequency of the clinical findings varies according not only to gender but also to the geographic regions. The PEDBD study group showed that children from European countries comparing to non-European counterparts are more likely to have articular, gastrointestinal, and neurologic findings. In addition, skin findings like acneiform lesions, pseudo folliculitis, and necrotic folliculitis are common in non-European children (12). In a recent study which is conducted with 205 of pediatric patients with BD from Turkey and Israel, necrotic folliculitis was more commonly detected in patients from Turkey than in patients from Israel (27).

## Etiopathogenesis and Genetic Background

The etiopathogenesis of Behçet's disease still couldn't be fully enlightened. The widely accepted opinion in the light of the current knowledge is that various identified and/or unidentified infectious and/or environmental triggers can take a role in individuals with genetic susceptibility (28). The disease is thought to have pathogenic mechanisms resembling autoimmune diseases, autoinflammatory diseases, and seronegative spondyloarthropathies (29, 30).

The relationship of infectious agents with the disease has been investigated since the definition of the disease, as much as that Hulusi Behçet himself also mentioned a possible viral etiology in the definition of the disease (31). There are several studies in the literature which are suggesting some of the microorganisms that may be a trigger for BD. One of these studies advocates that a cross-reaction detected between some of the streptococcal antigens and some of the heat shock proteins of human body is responsible for the pathogenesis of the disease (32, 33). In another study, the authors have reported that antibodies against some of the microorganisms such as *S. sanguinitis, S. pyogenesis*, are detected more frequently among Behçet patients than in the control group (32, 34). In addition, disease activation reported after oral interventions and significant differences shown in oral and intestinal microbiota of patients with BD in various studies also support the relationship between microbial agents and BD (32, 35–39). Yet, no objective causative relationship has been shown between single microorganism and BD.

One of the most frequently discussed topics is the genetic components of the disease. Human Leucocyte Antigen (HLA) B51 is the most widely known genetic predisposing factor for BD and its positivity increases the risk of development of BD by 5.78-fold (40). Males are more likely to have HLA-B51. Genital ulcers, ocular involvement and skin findings are more common in patients with HLA B51 (41, 42). The frequency of HLA B51 positivity among patients with BD and healthy populations is reported as 50–72% and 10–15%, respectively (2, 2, 26, 27). Therefore, the use of HLA-B51 for diagnostic purposes is controversial due to its high prevalence among healthy people.

Genome wide association studies (GWAS) have revealed associations between BD and several non-HLA genes like ERAP1, IL23 receptor (IL-23R), IL-23R/IL-12RB2, IL-10, STAT4 (32, 33, 43). ERAP-1, which has an epistatic interaction with HLA B51, takes an active role in the folding of the peptides that is required for the interaction between MHC-I molecules and peptides. It is shown that if the folding cannot be performed properly (misfolding), the IL23 / IL17 pathway may be activated (38–40). Some of the ERAP-1 polymorphisms have also been shown in patients with ankylosing spondylitis and psoriatic arthritis (44–46). In addition, the misfolding of HLA B27 in patients with ankylosing spondylitis, activation of IL23/IL17 pathway by some HLA-C molecules in psoriatic arthritis have been shown in several studies (47, 48). The MHC-1-opathy concept, which suggests that BD and spondyloarthropathies have similarities regarding immunopathogenic pathways, mainly arose from these findings (49).

Familial aggregation of Behçet's disease, which supports the disease's genetic background, has been shown in both children and adult patients with Behçet's disease (50, 51). It is also shown that the frequency of familial cases is significantly higher in pediatric patients than adult patients with BD (50). At least part of the higher frequency of familial cases reported in pediatric cases can be partly explained by the monogenic BD mimics described recently. Haploinsufficiency of A20 (HA 20) which is an excellent example of the monogenic mimics of BD, can be presented with clinical picture indistinguishable from BD (3).

In a recently published study, Manthiram et al. (52) reported the genetic similarities between recurrent aphthous stomatitis, BD and periodic fever, aphthous stomatitis, pharyngitis, adenitis (PFAPA) syndrome, and suggested grouping these diseases under “the Behçet related diseases” umbrella on a continuum like: recurrent aphthous ulcer as the mildest phenotype, PFAPA syndrome as a moderate form, and Behçet's disease as the most severe phenotype. In addition, Cantarini et al. (53) showed in their study conducted by applying PFAPA syndrome classification criteria to adult patients with BD according to their clinical findings during childhood that 30% of adult BD cases also met the PFAPA syndrome classification criteria in childhood. The authors suggested that the similar cytokine alterations may cause PFAPA syndrome during childhood and BD during adulthood. PFAPA syndrome is one of the most common periodic fever syndromes in childhood and its characteristic finding is oral aphthous lesions and fever episodes (54, 55). Despite its higher frequency in childhood, it is rarely reported in adulthood (56). In the light of aforementioned data, PFAPA syndrome which has genetic similarities and overlapping clinical findings with Behçet's Disease (oral aphthous lesion, fever), may reflect an early phenotype of BD. Further studies are needed to making solid conclusion for the relationship between BD and PFAPA syndrome.

## Clinical Findings

### Mucocutaneous Lesions

**Figure 1:**
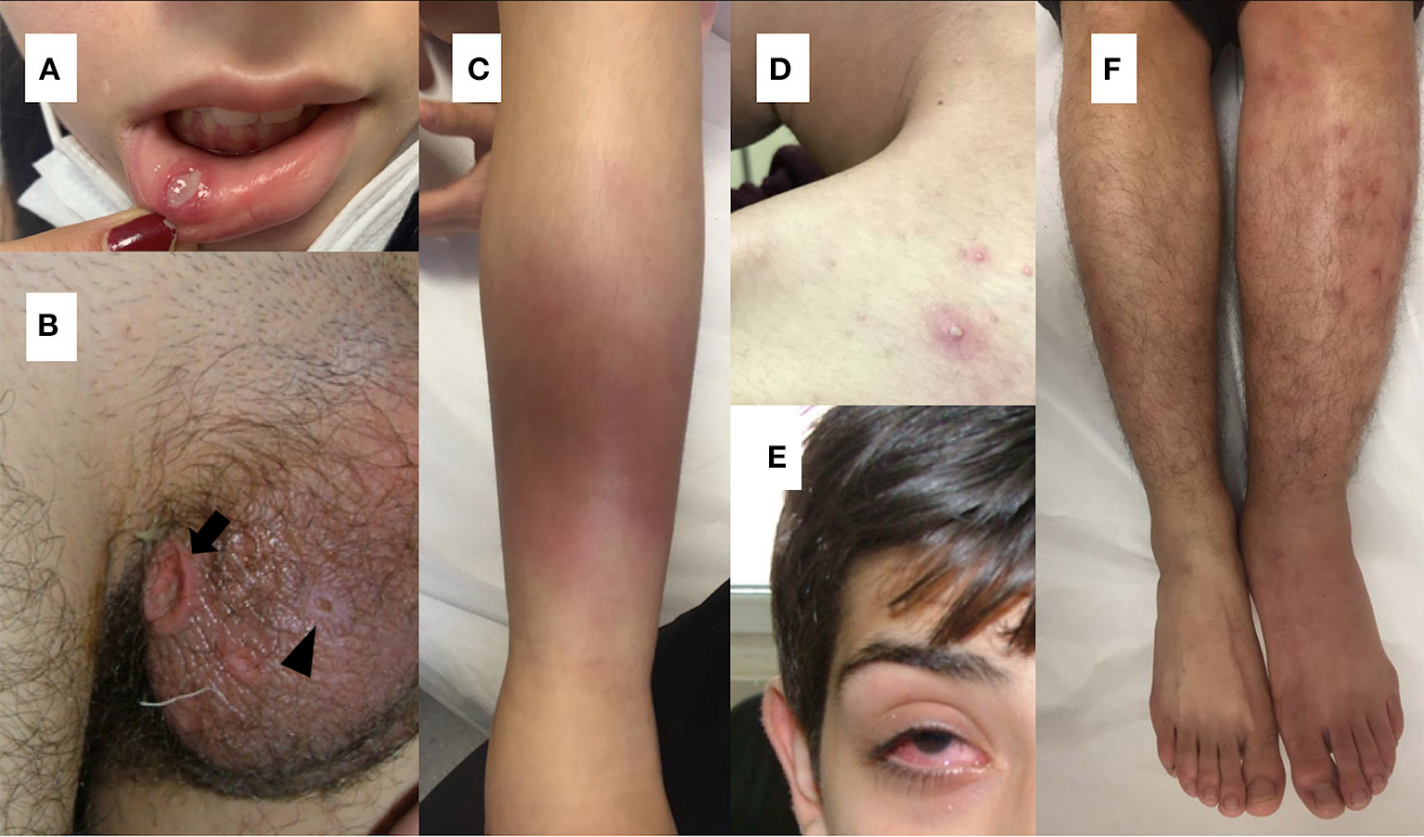
**(A)** Oral aphthous lesion **(B)** Genital ulceration (arrow) and scar (arrow head) **(C)** Erythema nodusum **(D)** Pustular lesion **(E)** Uveitis in a patient with Behçet's disease **(F)** Difference in diameter between extremities suggesting deep vein thrombosis in a patient with Behçet's disease.

As in adult patients with BD, recurrent oral ulcerations, which are seen in 96–100% of pediatric cases, are the most common finding of the disease in children (2, 21, 57–59). Non-scarring painful oral lesions are characterized by sharp circular shape with erythematous borders and usually occur on the tongue or on the oropharyngeal and buccal mucosa and can occur many years before the diagnosis has been established (4, 60, 61) (Figure 1A).

Genital ulcers which are reported in 57–93 % of adult cases, are usually located on scrotum or labia major and minor (26, 60) (Figure 1B). In contrary to oral lesions of BD, genital lesions tend to be painful, deeper, irregular, and heal with scarring (26). Children with BD are less likely to have genital ulcers than adult patients (58, 59, 62). Also, scarring is less common in pediatric patients (63).

The most commonly observed cutaneous lesions during the course of disease are erythema nodosum, papulopustular lesions, purpura, and folliculitis and these lesions have been reported in 37.3–66% of the pediatric cases (12, 21, 27, 58, 64, 65) (Figures 1C,D). Acneiform lesions have a distinct distribution being commonly located on the face, extremities, and trunk. This finding can help in establishing a differential diagnosis between acnes of BD and common acnes of adolescents (60).

The pathergy phenomenon is a nonspecific hypersensitivity reaction to trauma. It can be performed by puncturing the flexor aspect of forearm skin with a 20-gauge needle, and the test is considered positive if an indurated erythematous pustule develops at the site of trauma within 24 to 48 h (1). It should be kept in mind that the positive pathergy test is not pathognomonic to BD. A positive pathergy test should be accepted as a supporting finding or warning sign for BD. The rate of the pathergy test positivity has been reported as 14.5–80% among patients with BD from different populations (3, 58).

### Musculoskeletal Involvement

Musculoskeletal findings are reported in 20–40% of children with BD and can be seen as an early finding of the disease (2). Articular findings of BD are usually self-limited and heal without deformity. The most commonly affected joints are knee and ankle (66). Articular manifestations in BD can present as oligoarticular or polyarticular pattern and sacroiliac joint involvement and enthesopathy can also be seen. In a pediatric BD cohort, peripheral arthritis and axial skeleton involvement were detected in 47.4 and 16.6% of the patients, respectively (12).

### Eye Involvement

Eye involvement is one of the most important causes of morbidity in BD and is reported in 14.1–66.2% of pediatric BD cases (12, 21, 27, 58, 64, 65). Although it can develop at any time during the course of the disease, it most often appears within 2–3 years after the diagnosis (67). It has been reported that 10–20% of the adult patients have ocular involvement at the time of diagnosis (67). Koné-Paut et al. (68) reported that ocular involvement in children is less common than in adults yet has a more severe course. In contrast to this, there are also publications reporting that children are more likely to have ocular involvement (69, 70). Gallizzi et al. (65) reported that eye involvement (43.6%) was the second most common finding among their cohort of children with BD.

The most common findings detected in patients with ocular involvement are blurred vision, ocular pain, photophobia, and eye redness (71) (Figure 1E). Bilateral posterior uveitis is the most typical ocular involvement pattern of BD (2). Chronic bilateral non-granulomatous inflammation can affect both the anterior and posterior segments, causing pan-uveitis (23, 60). Anterior uveitis with hypopyon is one of the characteristic findings of ocular BD (60). Iridocyclitis, keratitis, episcleritis, vitreous hemorrhage, cataract, glaucoma, and retinal detachment can also be seen in ocular BD (71).

### Neurological Involvement

Neurological system involvement, namely neuro Behçet's Disease (NBD), is reported in 3.6–59.6% of the children with BD (12, 21, 58, 65, 68, 72, 73). NBD can be basically divided into two groups as parenchymal form and non-parenchymal vascular form. Although peripheral nervous system involvement can be seen, it is scarce in children (2, 74, 75). Parenchymal lesions generally tend to affect the brainstem, basal ganglia, spinal cord, and cerebral white matter (76). The main manifestations associated with the non-parenchymal vascular form are cerebral venous thrombosis and pseudotumor cerebri. The non-parenchymal vascular form is more common in children (4, 76).

Pediatric NPD has acute and progressive chronic presentations. Acute manifestations include recurrent aseptic meningitis and meningoencephalitis. Besides, acute onset headache, papillary edema, hemiparesis, ataxia, and epilepsy can also be seen. Chronic parenchymal manifestations which are usually irreversible, mostly involve neuropsychiatric conditions including memory loss, depression, anxiety and pseudobulbar syndrome (2).

### Vascular Involvement

Although BD can affect all sizes and types of vessels, venous system involvement is more common during the course of the disease (1). Vascular involvement in children is reported as 1.8–21%, and the most common vascular involvement in BD is lower extremity venous thrombosis (21, 58, 65, 68, 72). Deep vein thrombosis usually involves the iliofemoral veins, superior or inferior vena cava (Figure 1F). Dural venous sinuses and hepatic veins may also be involved (77). Embolism is not expected in thrombotic events seen in BD (2). Male gender and young age are reported as risk factors for vascular complications (24, 78).

Arterial involvement is reported in adult patients and pediatric cases as 3–12% and 1.8–14.7%, respectively (12, 21, 27, 58, 64, 65, 77, 79). Pulmonary artery aneurysm is the most common cause of mortality in BD (79). It has been reported that stenosis, pseudoaneurysm and occlusion in the arterial system can be seen in addition to pulmonary artery aneurysm (79).

### Gastrointestinal Involvement

Gastrointestinal (GI) system involvement in children with BD varies between 4.8 and 56.5% (4, 64). It has been reported that GI involvement is more common in children than in adults (23, 59). Gastrointestinal symptoms usually start within 4.5–6 years after the onset of oral ulcers (80). Although mucosal lesions may occur in any part of the digestive track, the ileocecal region is most frequently involved (4). The most common symptoms are abdominal pain, nausea, vomiting, dyspepsia, diarrhea, and gastrointestinal bleeding (60). It is difficult to differentiate the GI involvement of BD from inflammatory bowel diseases. The round ulcers, the focal single / multiple distribution patterns, <6 ulcers, and the absence of a cobblestone appearance were found to be related with BD (81). Intestinal ischemia due to arterial involvement and Budd-Chiari syndrome associated with venous involvement are other gastrointestinal manifestations (82, 83). The comparison of clinical and laboratory findings from major pediatric BD cohorts including more than 50 patients are presented in Table 1.

**Table 1:** Comparison of clinical and laboratory findings from major pediatric Behçet's disease cohorts including more than 50 patients.

	**Kone Paut et al. (12)**	**Shahram et al. (64)**	**Karincaoglu et al. (21)**	**Gallizzi et al. (65)**	**Atmaca et al. (58)**	**Butbul et al. (27)**
Number	156	204	83	110	110	*205*
Age of first symptom (years)	7.8 ± 4.3	10.5 ± 3.4	12.2 ± 3.5	8.34 ± 4.11	11.6 ± 3.4	11.08 (1-15.9)
Oral Aphthosis (%)	100	91.7	100	94.5	100	99.5
Genital Ulcers (%)	55.1	42.2	81.9	33.6	82.7	65.4
Cutaneous Signs (%)	66.6	51.5	51.8^*^	39.6	37.3^*^	48.8
Pathergy Positivity (%)	N/A	57	37.3	14.5	45.5	26.9
Ocular Sign (%)	45.5	66.2	34.9	43.6	30.9	14.1
Joint Involvement (%)	41	30.9	39.8	42.7	22.7	42.9
Gastrointestinal involvement (%)	29.4	5.9	4.8	42.7	-	13.2
Neurologic involvement (%)	59.6	4.4	7.2	30.9	3.6	14.6
Vascular (%)	14.7	6.4	7.2	1.8	3.6	10.7
Family History (%)	24.4	9.9	19	12	12.3	26.3
HLA-B51 positivity (%)	-	22.8	-	56.8	-	65.2

* *only erythema nodosum*.

## Main Differences Between Pediatric and Adult Onset Disease

The development of a full-blown disease phenotype takes longer in pediatric patients (5–8). Therefore, it should be kept in mind that some of the children with BD may not meet the classification criteria in the early stages of disease and such patients should be followed-up carefully for further clinical findings. It has also been reported that, while GI system involvement, neurological findings, arthralgia, and positive family history are more common in children, genital and vascular lesions are more common in adult patients (4, 5). It is shown that the frequency of familial cases is significantly higher in pediatric patients than adult patients with BD (50). In general a better disease outcome with lower severity score and activity index has been reported in children (4).

## Definitions and Classification Criteria

Behçet's disease, which is a multi-systemic vasculitis that can involve vessels of all sizes and types, was first described by Hulusi Behçet with the triad of oral aphthae, genital aphthae, and uveitis (1). While the term of “pediatrics BD” describes cases diagnosed in childhood, “juvenile BD” refers to cases that were diagnosed in adulthood but whose first symptoms started before the 16 years of age (68).

Many diagnostic and/or classification criteria for BD have been proposed so far (5, 9–11). The most frequently used are criteria proposed by the International Study Group (ISG) in 1990 (10). In 2014, a new set of criteria were proposed by the International Team for the Revision of the International Criteria (ICBD) (11). The main differences between the criteria of ICBD and the previous sets of criteria are that oral ulcers are not considered as a mandatory criterion in ICBD criteria, while the neurological and vascular findings are added. The sensitivity and specificity of these criteria in adult patients were reported as 96.1 and 88.7%, respectively (84).

Until now, the only set of criteria recommended for pediatric BD is the one by the Pediatric Behçet's Disease (PEDBD) study group (12). Pathergy testing is not included and an oral ulcer is not mandatory for diagnosis (12). Batu et al. (85) reported sensitivity and specificity as 52.9 and 100% for ISG criteria and 73.5 and 97.7% for PEDBD criteria, respectively. In a recently published study, Ekinci et al. (86) found sensitivity and specificity as 87.5% and 100% for ISG criteria, 93.7 and 98.1% for ICBD criteria, and 93.7% and 96.2 for PEDBD criteria, respectively. Main diagnostic/classification criteria proposed are represented in Table 2.

**Table 2:** Main diagnostic/classification criteria for Behçet's disease.

**Criteria of the International study group for BD (10)**
***Recurrent Oral Ulceration (Mandatory):*** Minor aphthous, major aphthous, or herpetiform ulceration observed by physician or patient, which recurred at least 3 times in one 12-month period
**Plus 2 of**
***Recurrent genital ulceration:*** Aphthous ulceration or scarring, observed by physician or patient
***Eye lesions:*** Anterior uveitis, posterior uveitis, or cells in vitreous on slit lamp examination; or retinal vasculitis observed by ophthalmologist
***Skin lesions:*** Erythema nodosum observed by physician or patient, pseudo folliculitis, or papulopustular lesions; or acneiform nodules observed by physician in post adolescent patients not on corticosteroid treatment
***Positive pathergy test:*** Read by physician at 24–48 h.
**International Criteria for Behçet's Disease** **(****11****)**
• Ocular lesions 2 points • Genital aphthosis 2 points • Oral aphthosis 2 points • Skin lesions 1 point • Neurological manifestations 1 point • Vascular manifestations 1 point • Positive pathergy test^*^ 1 point^*^ *4 or more points are required for diagnosis*.
^*^Pathergy test is optional. If it is performed and resulted as positive, additional 1 point may be added.
**Pediatric Criteria for Behçet's Disease** **(****12****)**
**Recurrent oral aphthosis:** At least three attacks/year
**Genital ulceration or aphthosis:** Typically, with scar
**Skin involvement:** Necrotic folliculitis, acneiform lesions, erythema nodosum
**Ocular involvement:** Anterior uveitis, posterior uveitis, retinal vasculitis
**Neurological signs:** With the exception of isolated headaches
**Vascular signs:** Venous thrombosis, arterial thrombosis, arterial aneurysm
*At least 3 criteria are required for the diagnosis*.

## Differential Diagnosis

As Behçet's disease can present with various combinations of clinical symptoms related to several organ systems, its differential diagnosis is extensive. Recurrent oral aphthae, the most common manifestation of pediatric BD, are nonspecific and can also be seen in various infections (herpes simplex, syphilis, HIV), vitamin deficiencies, hematological diseases like cyclic neutropenia, PFAPA syndrome, hyper immunoglobulin D syndrome, systemic lupus erythematosus, and inflammatory bowel disease (60, 66). Oral aphthae tend to be multiple and usually locate on the oropharynx, buccal mucosa, and heal without scarring. Main et al. (61) suggested that multiple ulcers, variable-sized ulcers with erythematous borders, and ulcers on the soft palate and oropharynx are the features that may be beneficial for differencing BD-related oral ulcers. Genital ulcers should be differentiated from venereal diseases like syphilis, herpes simplex virus especially in the sexually active adolescents (87). Overlapping features with BD such as constitutional manifestations, oral aphthae, non-erosive arthritis, neurologic, and vascular findings can be seen in the course of systemic lupus erythematosus. Similarly, ANCA-related diseases and BD also have common clinical manifestations, including constitutional, musculoskeletal, vascular, eye, and skin manifestations (88). These diseases should be kept in mind in the case of these common findings in the differential diagnosis of BD, and antibody testing may be beneficial for differencing these diseases. Inflammatory bowel diseases (IBD) are another excellent example of the diseases among differential diagnoses of BD. Oral ulcers, non-erosive arthritis, gastrointestinal involvement, eye and skin manifestations are typical findings of inflammatory bowel diseases. Especially gastrointestinal findings of BD and IBD are hard to distinguish, and histologic confirmation is usually needed to make a diagnosis (88). Sarcoid (oral ulcers, lung disease, erythema nodosum, neurological complications, skin manifestations) and SAPHO syndrome (arthritis, acne, pustular lesions) should be included to the differential diagnosis of BD. Mouth and genital ulcers with inflamed cartilage syndrome (MAGIC syndrome) is a syndrome with symptoms of both BD and relapsing polychondritis (88). Oral and genital ulcers, skin findings, nonerosive arthritis, neurological involvement can be seen in the course of some autoinflammatory diseases. Haploinsufficiency of A20, one of autoinflammatory diseases, should be noted as an example of the monogenic mimickers of BD. Haploinsufficiency of A20 is caused by autosomal dominant mutations in TNFAIP3 gene. Oral and genital ulceration, skin manifestations, recurrent fever, gastrointestinal manifestations, arthritis and eye findings are common findings of HA20, and the disease can be presented with clinical picture indistinguishable from BD. Therefore, HA20 should be remembered in familial cases (3).

## Treatment

There are no published internationally approved recommendations regarding the management and treatment of pediatric Behçet's disease. In addition, our knowledge about treatment response in Behçet's disease is mainly based on studies conducted in adult patients with BD. Therefore, many pediatric rheumatologists follow EULAR Behçet's disease management recommendations (89).

The primary goal for the treatment is preventing the organ damages by suppressing the ongoing inflammation and forestalling the disease flares. The treatment of the BD can be onerous due its multisystemic nature and a multidisciplinary approach is essential for management of the patients. Due to the extensive distribution of the disease, the management of BD should be made with a multidisciplinary approach (89, 90).

### Conventional Treatments

Corticosteroids, which can be used via topical, oral and intravenous route, have strong and rapid anti-inflammatory effects (71, 89). Topical corticosteroids (triamcinolone acetonide cream) are initial treatment for oral and genital aphthous lesions, and they can also be used for anterior uveitis (91). Topical sucralfate can also be tried for oral and genital ulcers alone or in combination with topical corticosteroids (92, 93). Systemic steroids are effective in most of the clinical manifestations of BD. Systemic steroids, which are preferred to use in combination with other anti-inflammatory drugs, are recommended for oral or genital lesions unresponsive to topical treatments and cutaneous lesions resistant to colchicine (90, 94). They are also used for thrombotic events like acute DVT and cerebral venous thrombosis, arterial involvement and severe gastrointestinal involvement. Pulse methyl prednisolone is recommended for acute sight threatening uveitis for 1–3 days (91). Due to their severe side effect profile, high doses and prolonged use of steroids as a monotherapy should be avoided (71, 89, 90).

Colchicine is an anti-inflammatory drug acting by inhibiting neutrophil migration (71). It is recommended as a first line treatment for preventing of mucocutaneous lesions (89, 95). It is also shown that colchicine is effective in decreasing the number of the affected joints after 2 years follow-up (96).

Azathioprine is another immunosuppressive agent that is widely used in management of the BD. It is recommended for patients with severe mucocutaneous manifestations, persistent arthritis, deep venous thrombosis, active posterior uveitis, or isolated anterior uveitis (in males), GI and neurologic involvement (66, 82, 89–91, 97).

Methotrexate can be used for ocular and mucocutaneous findings of the disease. Also, it is an option for combination therapy in neuro-Behçet's disease (66).

Cyclosporine A is mainly recommended for patients with severe ocular involvement and persistent mucocutaneous lesions (92). Patients using cyclosporine A should be monitored for severe side effects like hypertension, renal failure, or neurologic findings (71, 98).

Cyclophosphamide is another therapeutic option for patients with BD. It is usually used for severe manifestations of the disease such as pulmonary artery involvement, Budd-Chiari syndrome, parenchymal neurologic involvement, etc. (89, 95).

### Biologic Treatments

Main anti-TNF agents used in pediatric patients are etanercept (TNF receptor p75 fusion protein), adalimumab, and infliximab (monoclonal antibody). These agents are recommended to be used in patients who cannot be controlled by conventional immunosuppressive treatments or in cases with intolerance or allergic reactions to conventional agents (89, 95). Anti-TNF agents have been shown to provide a significant reduction in steroid dose (94, 99). These agents are recommended as the first-line treatment for patients with severe neuro Behçet disease (89). Also, it is shown that these agents are effective in the treatment of refractory deep venous thrombosis and arterial involvement. Many studies are reporting that infliximab is highly effective in severe eye involvement (67, 99, 100). Besides, infliximab and adalimumab are stated to be effective in moderate and severe GI Behçet's disease (101).

Interferon-alpha (IFN- α) is a cytokine with immunomodulatory effects, and it has been shown that IFN-α is effective in the treatment of resistant posterior uveitis (102). Besides, partial remissions have been reported in mucocutaneous symptoms with IFN α−2a or α−2b (102).

Anakinra, canakinumab, tocilizumab, ustekinumab, secukinumab, apremilast, and mycophenolate mofetil have also being tried in adult patients with BD (103, 104). Yet, these agents are not used routinely in pediatric cases and further studies are needed for their efficacy and the safety in pediatric cases.

Intravitreal corticosteroid injection is another therapeutic intervention for severe ocular involvement especially in the case of unilateral exacerbation (91).

## Conclusion

In conclusion, Behçet's Disease (BD) is a rare systemic vasculitis with unique geographic distribution around the historical silk road. The pediatric form of the disease differs from the adult-onset BD in several aspects like the frequency and distribution of clinical findings, disease severity, and outcome. The diagnosis of pediatric BD is often challenging because of its frequent incomplete clinical picture. The differential diagnosis is extensive and monogenic mimickers of the disease such as A20 haploinsufficiency should be kept in mind in pediatric cases. There is a need for clinical trials will be conducted with pediatric cases, and evidence-based treatment/management recommendations for pediatric BD.

## Author Contributions

OKa, MY, and FH: concept and design. AA, SS, KB, and OKo: supervision. MY, OKo, FH, AA, SS, and KB: literature search. MY, FH, and AA: writing manuscript. AA, SS, KB, and OKa: critical review. All authors contributed to the article and approved the submitted version.

## Conflict of Interest

The authors declare that the research was conducted in the absence of any commercial or financial relationships that could be construed as a potential conflict of interest.
